# New Active Packaging Based on Biopolymeric Mixture Added with Bacteriocin as Active Compound

**DOI:** 10.3390/ijms221910628

**Published:** 2021-09-30

**Authors:** Camila Ramão Contessa, Gabriela Silveira da Rosa, Caroline Costa Moraes

**Affiliations:** Science and Engineering of Materials Graduate Program, Federal University of Pampa, 1650, Maria Anunciação Gomes Godoy Avenue, Bagé 96413-170, RS, Brazil; camilaramao@hotmail.com (C.R.C.); gabrielarosa@unipampa.edu.br (G.S.d.R.)

**Keywords:** active packaging, barrier property, *Lactobacillus sakei*

## Abstract

The objective of this work was to develop a chitosan/agar-agar bioplastic film incorporated with bacteriocin that presents active potential when used as food packaging. The formulation of the film solution was determined from an experimental design, through the optimization using the desirability function. After establishing the concentrations of the biopolymers and the plasticizer, the purified bacteriocin extract of *Lactobacillus sakei* was added, which acts as an antibacterial agent. The films were characterized through physical, chemical, mechanical, barrier, and microbiological analyses. The mechanical properties and water vapor permeability were not altered by the addition of the extract. The swelling property decreased with the addition of the extract and the solubility increased, however, the film remained intact when in contact with the food, thus allowing an efficient barrier. Visible light protection was improved by increased opacity and antibacterial capacity was effective. When used as Minas Frescal cream cheese packaging, it contributed to the increase of microbiological stability, showing a reduction of 2.62 log UFC/g, contributing a gradual release of the active compound into the food during the storage time. The film had an active capacity that could be used as a barrier to the food, allowing it to be safely packaged.

## 1. Introduction

Bioplastics have attracted the attention of researchers as a possible alternative to plastic products from petrochemical and non-renewable sources, which are responsible for several environmental problems, especially associated with difficult degradation [1]. These alternative and ecologically correct materials are important for sustainable growth because they are produced from renewable resources [2].

Many biopolymers have been used in the elaboration of bioplastics. Agar-agar is a biopolymer belonging to the natural polysaccharides extracted from red algae, of the *Rhodophyta* class, being the structural carbohydrate of the wall of these cells [3]. It is a thermoplastic, biocompatible, and biodegradable material, widely used in several industrial branches. Bioplastic films based on this polymer, however, are brittle in nature, have poor mechanical properties and high sensitivity to water because they are hydrophilic in nature, thus limiting its application in a product of high humidity [4].

Chitosan is derived from chitin, obtained mainly from residues of crab and shrimp shells, however, it can be obtained from several other sources such as cell wall and cell membrane of fungi, exoskeleton of insects, and arachnids [5]. The deacetylation of chitin occurs from the transformation of acetamide (NHCO_3_) into amine (NH_2_) in basic medium. They can be produced with different degrees of deacetylation and molecular weights, which directly influence the properties of bioplastics such as viscosity, solubility, tensile strength, and elongation [6,7]. Chitosan has some disadvantages for applications in bioplastic films because it presents low solubility, not allowing the interaction with other compounds often used for the elaboration of a film [5].

In this way, the union of these two polymers allows for the synthesis of a bioplastic film with appropriate characteristics for application in the food industry, since the hydrophobicity of chitosan allows the application in products with high water activity, and the thermal characteristics of agar-agar allow the integrity of the material in relation to the temperatures used in the sterilization processes, for example [8,9].

Research has been undertaken on developing new materials that aim to expand the potential for the application of bioplastics in the packaging industry. An alternative is the addition of active compounds in the biopolymeric matrix. The main objective of the bacteriocin extract is to add antimicrobial characteristics and contribute to the synthesis of a material with adequate characteristics for the storage of a food product, acting in the protection and prolonging the useful life. The use of bacteriocins is interesting, since they are a protein nature that show biopreservative potential. Its characteristics include antimicrobial action with specific action, not altering the intestinal microbiota, as they are digested by intestinal enzymes such as trypsin and pepsin, have a broad antimicrobial spectrum, and act in the inhibition of pathogenic bacteria and food spoilage [10].

The aim of this study was the development of an active bioplastic film from a polymer blend, incorporated with a purified bacteriocin natural extract. The union of two polymers allows the synthesis of unique properties, acting in the improvement of specific flaws, originating from a single material. The novelty of this work is based on the establishment of the best proportion of the chitosan/agar-agar and plasticizer union in the solution for the synthesis of a bioplastic film, the use of antimicrobial extract of a new bacteriocin obtained from the food matrix, and the study in situ for feasibility of application as an active packaging.

## 2. Materials and Methods

### 2.1. Materials

Chitosan (Oakwood Chemical) molar mass 170.7–198.5 kDa, degree of deacetylation 95% according to the manufacturer’s data and agar-agar (Himedia, WF, Pelotas, RS, Brazil) were used as components forming the film. Glycerol (Alphatec, WF, Pelotas, RS, Brazil) was used as a plasticizer to improve matrix flexibility. For the chitosan dilution, acetic acid (Synth) 1 M was used. Bacteriocin extract of *Lactobacillus sakei* obtained from food matrix, purified by precipitation of 80% ammonium sulfate, supernatant fraction was used as an antibacterial agent. Agar Mueller Hinton (Himedia) was used for the analysis of disc-diffusion and microatmosphere.

### 2.2. Bioplastic Film Preparation

The filmogenic solution was obtained by adding agar-agar, chitosan, and glycerol in the proportions established in the treatments (Table 1) of the central composite rotational design (CCRD, 2^3^), according to Rodrigues and Iemma [11]. The experimental design aimed at identifying the influence of the formulation on film properties (TS: tensile strength and EB: elongation). The determined ranges were obtained from preliminary tests. Chitosan was dissolved in 1 M acetic acid for 24 h at 25 °C and, subsequently, stirred for 30 min. The agar dissolved in distilled water. The film-forming solution (50 mL) was poured into a 15 cm diameter Petri dish. The films were obtained by the casting method, the components were solubilized with magnetic stirring in a heating plate at 80 ± 3 °C (with the exception of chitosan), and evaporated in a convective dryer for 24 h at 40 ± 2 °C. The films were conditioned in a desiccator with sulfuric acid solution, at a relative humidity (RH) of 50% for 48 h before carrying out the analyses.

The bioplastic films obtained in the optimized condition had the incorporation of purified bacteriocin extract (liquid form), in its formulation as a partial solvent substitute (10%), this value being defined in preliminary tests. All analyses were done in triplicate and the result was expressed in mean and standard deviation.

### 2.3. Bacteriocin Extract

The *Lactobacillus sakei* strain was previously isolated by the research group and cryopreserved at −14 °C. The activation used *Man, Rogosa,* and *Sharpe* broth (MRS) at a temperature of 32 °C and agitation of 150 rcf for a time of 48 h. After the fermentation process, centrifugation was performed for 5500 rcf/15 min to remove the cells, since bacteriocins are extracellular compounds, thus obtaining the cell-free extract [12].

The cell-free extract was purified by the ammonium sulfate precipitation method, where it was added under agitation to saturation of 0% to 80% of this same salt, where it remained under refrigeration for 24 h, after this time of dormancy, it was centrifuged at 5500 rcf/4 °C for 15 min.

### 2.4. Bioplastic Films Characterization

#### 2.4.1. Thickness

The film thickness was measured with a digital pachymeter (DIGIMESS 0.01 mm) at fifteen random locations on the film.

#### 2.4.2. Mechanical Properties

The tensile strength at break (TS) and elongation at break (EB) were performed in a texturometer (TA.XP2i, SMD, GBR) according to the American Society for Testing and Materials D 882-12 [13] standard method. Samples were prepared with rectangular geometry 100 mm long and 25 mm wide and conditioned at 25 °C in 50% relative humidity for 48 h prior to analysis. The initial grip separation was set at 50 mm and the speed at 50 min/mm. The measurements were performed three times, and the average was obtained. The calculation of tensile strength was found from Equation (1), followed by the calculation of elongation at break represented by Equation (2).

(1) TS=FM/A

where *TS* is tensile strength (MPa); *FM* is the maximum force at the time of the film break (N); and *A* is cross-sectional area (m^2^).

(2) $$EB=\left(DR/\;DI\right)\;\times \;100$$

where *EB* is represented by the elongation (%); *DR* by the distance at the moment of rupture (cm); and *DI* is initial separation distance (cm).

#### 2.4.3. Water Vapor Permeability

The permeability was determined gravimetrically by the standard method E96/E96M-14 [14], where the films were positioned in capsules containing anhydrous calcium chloride (CaCl_2_). The set was conditioned in a chamber with a relative humidity of 50% being weighed after seven days to determine the permeability of the films to water vapor from Equation (3).

(3) $$wvp=\left(MP.L\right)/\left(t.A.∆P\right)$$

where wvp is the permeability to water vapor (kg/Pa·s·m); *MP* is the mass of moisture absorbed (kg); L is the thickness of the film (m); *t* is the analysis time (s); *A* is the area of the exposed surface of the film (m^2^); and ∆P is the water partial pressure difference through the film (Pa).

#### 2.4.4. Water Solubility

Samples of 2.5 cm in diameter of the films were used to determine solubility in water. Initially, the initial dry mass of the films was determined in a 105 °C oven for 24 h. The samples were then immersed in 50 mL of distilled water and subjected to orbital agitation of 175 rcf for 24 h at a temperature of 25 °C. After further drying, the final dry mass was determined, thus the solubility of the films was expressed from Equation (4) [15].

(4) $$\mathrm{SW}=\left(\left(MI-MF\right)/100\right)\times 100$$

where *SW* the (%) of solubility in water; *MI* is the initial dry mass (g); and *MF* is the final dried mass (g).

#### 2.4.5. Swelling Property

Films cut into samples of 2.5 × 2.5 cm were used as a specimen, which were weighed and immersed in ultrapure (miliQ 25 °C) water for 2 min. The wet samples were cleaned with paper towels to absorb the excessive moisture and then weighed again. Measurements were repeated three times and an average was taken as a result. The amount of water absorbed was calculated using Equation (5) [16].

(5) $$Swelling\;\left(\%\right)=\left(100\left(M2-M1\right)\right)/M1$$

where *M*1 and *M*2 are the masses (g) of the samples wet and dried, respectively_._

#### 2.4.6. Moisture Content

The 2.5 cm × 2.5 cm specimens were weighed (m1) and dried at 105 °C/24 h, reweighed (m_2_), and the moisture content (WC) determined (Equation (6)) as the percentage of water lost during drying and reported on a wet basis [16]. The measurements were repeated three times and an average was taken as a result.

(6) $$WC\;\left(\%\right)=\left(100\left(M1-M2\right)\right)/M1$$

where *M*1 and *M*2 are the masses (g) of the samples wet and dried, respectively_._ These were repeated three times and an average was taken as a result.

#### 2.4.7. Color, Transmittance, and Opacity

The color of the films was determined by a colorimeter (Minolta^®^ CR-300) using white as the standard (L* = 99,36; a* = −0,12; b* = −0,07). The values of (L*), (a*), and (b*) were used to characterize the color of the film on the Hunter Lab scale. The total color difference (Δ*E*) was calculated using Equation (7) [17].

(7) $$\Delta E={\left({\left(Li^{*}-L^{*}\right)}^{2}+{\left(Ai^{*}-a^{*}\right)}^{2}+{\left(Bi^{*}-b^{*}\right)}^{2}\right)}^{\frac{1}{2}}$$

Transmittance and opacity were determined from SP 220 to 600 nm spectrophotometer readings, the 1 × 4 cm long specimens were inserted in a glass bucket and the transmittance and absorbance readings were performed. The opacity was calculated using Equation (8) [17].

(8) $$O=\left(Abs_{600}\right)/L$$

where *O* is the opacity; *Abs*_600_ is the absorbance value at 600 nm; *L* is the thickness of the film (mm); $Li^{*}$ refers to the brightness of the standard color (white) and $L^{*}$ the luminosity of the sample; $Ai^{*}$ indicates the red-green hue of the standard color and $a^{*}$ the same shade as the sample; and $Bi^{*}$ represents the yellow-blue shade of the standard color and $b^{*}$ of the sample.

#### 2.4.8. Fourier Transform Infrared Spectroscopy (FTIR)

The film infrared spectra were characterized by Fourier transform infrared spectroscopy (FTIR) with spectrophotometer model FTIR-8400S (Shimadzu, Japan), combined with the ATR accessory.

#### 2.4.9. Surface Area and Mean Pore Diameter

The adsorption technique (Quantachrome Instrument, NOVA 4200e, Boynton Beach, FL, USA) and the BJH method (Barret, Joyner and Halenda) were used to obtain the characteristics surface area and mean pore diameter of biofilms. Initially, the samples were kept at 77.35 K for 72 h in vacuum for degasification [18]. 

#### 2.4.10. Thermal Stability 

Thermal decomposition was evaluated by thermogravimetric analysis (TGA) using about 4.5 mg of sample in the instrument (SHIMADZU TGA 50, Kyoto, Japan) at a temperature range of 25 to 600 °C with a heating rate of 10 °C·min^−1^ and N_2_ flow rate of 50 mL·min^−1^.

#### 2.4.11. In Vitro Antibacterial Property

In order to evaluate the antibacterial properties, the adapted disc-diffusion method described by NCCLS [19] was used for direct contact. Mueller Hinton Agar, inoculated with the indicator strains *Escherichia coli* (ATCC 11229), *Staphylococcus aureus* (ATCC 12598), *Listeria monocytogenes* (ATCC 7644), and *Salmonella enteritidis* (ATCC 13076) separately, was used as the culture medium. Contamination occurred by spreading the standardized bacterial culture with approximately 1.5 × 10^8^ CFU/mL on the surface (200 µL). Contact was made using 6 mm diameter discs for film without extract (FSE), film with extract (FCE), and also 10 µL of the purified supernatant fraction extract (EPS) of bacteriocin. The plates were refrigerated for 1 h in order to migrate the bacteriocin to the culture medium, similar to [20], and incubated at 35 °C/24 h. After the incubation period, the diameter of the inhibition zone was measured with a digital pachymeter.

For the microatmosphere analysis, the same media and indicator cultures were used, but the films were inserted in the lid of the Petri dish and these were incubated inverted and wrapped with Parafilm in order not to have detachment of vapors, thus providing a controlled microatmosphere [21]. 

#### 2.4.12. Bioactive Release

The release profile was determined by immersion, (0.9 g of the films were submerged in 10 mL of 60% glycerol solution) for 20 days, being analyzed on days (1, 2, 3, 4, 5, 10, 15, and 20). The release was established from antibacterial analyses based on NCCLS [19] methods, with SP 220 to 625 nm spectrophotometer readings, using standardized bacterial culture with approximately 1.5 × 10^8^ CFU/mL of *Escherichia coli* (ATCC 11229) and *Staphylococcus aureus* (ATCC 12598). The 60% glycerol solution was used as a simulator of an aqueous food system with water activity between 0.7 to 1.0 [22,23].

### 2.5. Application In Situ

To simulate the use of the film applied to food, the films with and without extract, were adhered to packages of Minas Frescal cream cheese, replacing the aluminum membrane traditionally used in this type of product. This way, thermotolerant coliforms and positive coagulase staphylococci were analyzed on the 7th, 14st, and 21th accompanying day, in this time, the product was stored inverted so that it would come into contact with the films, at cooling temperature.

An intentional contamination was also made to the cream cheese with 10^6^ CFU/of *Staphylococcus aureus* and *Escherichia coli*, separately. The microorganisms analyzed were thermotolerant coliforms and positive coagulase staphylococci performed by the traditional method of multiple tubes, in accordance with the American Public Health Association (APHA) and direct plate count method of APHA, respectively [24].

### 2.6. Statistical Analysis

All data collected were presented with mean ± standard deviation and statistically analyzed by one-way ANOVA and Tukey’s later test with a 95% confidence level. For experimental planning, the effects of significant (independent) variables for TS and EB were identified using the Pareto diagram with a 90% confidence interval. Analysis of variance (ANOVA) and Fisher’s Test were used. To assess the significance and quality of the mathematical models generated. The optimization of the formulation was performed using the desirability function to maximize the properties of bioplastics.

## 3. Results and Discussion

### 3.1. Optimization of Bioplastic Formulation

The results obtained in the study of the formulation of bioplastic films through experimental design are presented in Table 2.

From the results, we can see the great variability of responses between treatments, indicating the influence of independent variables on the evaluated responses. The central point (treatment 15, 16, and 17) presented a small variation, showing the reproducibility of the process of obtaining the films. The values obtained for TS and EB varied between 1.31 to 11.15 MPa and 14.90 to 51.03%, respectively, and are in accordance with the ranges found by Andonegi et al. [25], who studied chitosan–collagen blends with values around 11.03 MPa and 19.7% and Moradi et al. [26], who obtained rupture tension values between 8.73 to 14.11 MPa when evaluating chitosan–polyethylene films. Jridi, Abdelhedi, Zouari, Fakhfakh, and Nasri [27] also obtained similar values for elongation at rupture when studying the interaction of compounds such as Arabic agar and gelatin-agar, where the values varied from 7.59 to 27.25%.

**Figure 1:**
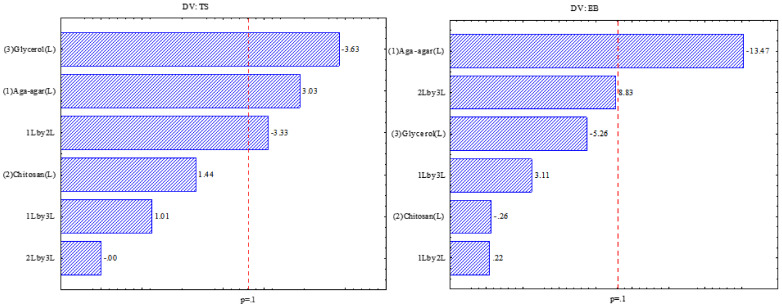
Effect analysis for tensile strength (TS) and elongation at break (EB).

Figure 1 indicates that for the tensile strength response the effect of the glycerol, agar-agar, and chitosan variables was significant, indicating that with the increase of plasticizer the resistance is decreased. The same was observed by Chevalier, Assezat, Prochazka, and Oulahal [28] in which films with 13.2% glycerol had TS between 16 and 38 MPa and films with a higher amount of plasticizer (24.2% glycerol) had TS between 2 and 13 MPa, which is due to the interaction between water and glycerol molecules, which leads to decreased intermolecular interactions, increasing the formation of vacant spaces and consequently decreased mechanical resistance [29]. The higher the agar-agar proportion, the greater the tensile strength, due to the increase of the hydrogen bonds between the molecules, producing a more compact structure [30,31]. 

For the elongation at rupture, the negative effect of the agar-agar variable was significant, indicating that when the agar-agar concentration increases, the elongation is reduced. Wu, Geng, Chang, Yu, and Ma [31], when studying the effect of agar concentration in potato starch films, observed that the addition of agar-agar up to 5% in relation to the amount of starch contributed to the increase in elongation, with higher concentrations of agar observed with a decrease in elongation capacity. With lower concentrations, the agar allows the absorption of moisture, which decreases the hydrogen bonds, increasing the mobility of the film and increasing the elongation. However, at higher concentrations, agar acts in the formation of bonds between hydrogen and the polymer, restricting the mobility of the polysaccharide chains and consequently an increase in elongation.

The analysis of variance (ANOVA) with a 90% significance level per residual SS was performed and the results are shown in Table 3.

It was possible to confirm the fit of the mathematical statistical models to the data obtained and their significance (R^2^ > 0.8 and Fcal > Ftab) (Equations (9) and (10)). The squares express the coded values of the model

(9) $$TS=7.61+1.51\left(A\right)-1.24{\left(C\right)}^{2}-1.81\left(G\right)-1.66\left(A\right)\left(C\right)$$

(10) $$EB=22.22-6.73\left(A\right)+4.16\;{\left(A\right)}^{2}$$

where *(A*) is agar-agar, (*C*) chitosan, and (*G*) glycerol.

**Figure 2:**
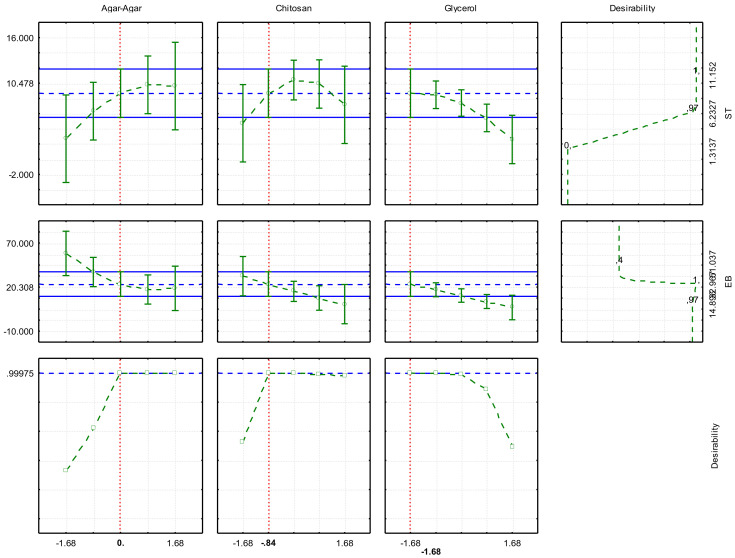
Desirability function (TS and EB optimization).

The optimization of experimental conditions to obtain films with better responses, TS, and EB was performed through the desirability analysis (Figure 2). 

According to the profile analysis, a desirability of 0.99 between a scale of 0 to 1 was obtained. The optimization considering the real variables was: 0.50 g, 0.49 g, and 0.15 g for agar-agar, chitosan, and glycerol, respectively, indicating the obtaining of films with 10.48 MPa of TS and 20.31% of EB. After playing the movie in the conditions indicated by the optimization and characterization, the responses found were 13.57 ± 2.17 MPa and 15.51 ± 2.87%, respectively. Both results can be considered similar to those predicted in the model, resulting in a bioplastic with the best characteristics according to the studied variables. The values found indicate good characteristics, being suitable in handling the packaging, allowing it not to break when wrapping the food, and to remain intact during transport, packaging, and handling.

### 3.2. Characterization of Active Bioplastic Film 

The results of the mechanical properties are illustrated in Table 4. 

TS and EB did not present a significant difference (*p* < 0.05) for films with and without the extract, which indicates that the extract did not interfere significantly in the mechanical resistance of the film. According to Pastor, Sánchez-González, Chiralt, Cháfer, and González-Martinéz [32], this property depends on the composition of the film, especially the concentration of polymers, which was not changed for the synthesis of ESF and FCE, which explains this behavior. This result indicates that there was possibly the interaction of all constituents used in the synthesis of bioplastics. Sánchez-González, Cháfer, González-Martínez, Chiralt, and Desobry [33] attributed that to the addition of active compounds being able to provide structural discontinuities in the material due to the immiscibility of the constituents, leading to a decrease in intramolecular forces and consequently a reduction in mechanical properties. The results differed from [15,34,35], who reported a reduction in mechanical properties of films added of other compounds (extracts) in relation to the control film.

The TS results of the FCE and FSE films were slightly higher than those of [36] of incorporated chitosan with 1.5% grapefruit seed extract, which showed a tensile strength of 8.93 MPa. For the EB response, the films showed lower results than the green tea extract embedded agar–gelatin films prepared by Giménez, Lacey, Santín, López-Caballero, and Montero [37], where they obtained 59% elongation, however, the handling of both was satisfactory.

Table 5 presents the results of the physical, barrier, and chemical characterization for films produced with and without extract.

The thickness of the films showed a significant difference between the FCE and FSE control film due to the addition of solids present in the extract. Riaz et al. [17] explained that thickness could also be changed due to the molecular interaction of polymers with the extract, and there may be short distance bonds, resulting in a more compact structure and consequently greater thickness. Similar findings were described by Kanmani and Rhim [38] in agar-based films incorporated with grapefruit seed extract, which obtained films in the range of 0.036 to 0.053 mm and Kaya et al. [39] in the range of 0.045 to 0.099 mm in chitosan films with fruit extract and *Berberis crataegina* seed oil. 

The water vapor permeability (Table 5) did not present significant difference between the FCE and FSE films. Both materials presented lower values than those reported by [40,41] on agar-agar films reinforced with nanocrystalline and nanobacterial pulp, respectively, Hankar and Rhim [42] on agar-agar and Reddy composite films; and Rhim [43] on agar and blackberry pulp nanocellulose films, which is a promising result. When water vapor permeability is favored in food products, they generally have a shorter lifespan due to the transfer between the external environment and the product, thus the use of bioplastic films aims at reducing this transfer. 

The results for water solubility indicate a significant difference between films with and without the extract. The film with the extract had twice the solubility, possibly due to its hydrophilic characteristics, similar to what occurred with Narasagoudr, Hegde, Chougale, Masti, and Dixit [44] in chitosan and poly(vinyl alcohol) films. However, the values were lower when compared to [45,46] in gelatin and chitosan films and Wang and Rhim [47] in agar-agar, alginate, and collagen films, which presented films with solubility around 43.45%, 58.13%, and 63%, respectively. This is due to the presence of chitosan in the formulation, which has hydrophobic behavior. The solubility of the bioplastic film is important when an application in food is intended, especially when the food presents high content of humidity, which is the desired application in this study.

The property of swelling is the capacity of the film to retain water and the lower this capacity, the more effective the barrier of the food with the external environment. The values obtained indicate that there has been a significant decrease in the swelling property of the film containing the bacteriocin extract in relation to the control film. The values found in this study were below those found by Wang and Rhim [47] and [16] in agar-agar films with 2363.7% and gelatin with 400%, respectively, and similar to those found by Riaz et al. [17] in chitosan films with Chinese chives root extract, where they also observed a reduction in the degree of swelling with the addition of the extract from 57.38% to 40.49%, explained by the presence of hydroxyl (hydrophilic groups) in the chitosan molecule.

The visual characteristics of a package are attractive to the eyes of the consumer, which are often influenced by the color and transparency of the materials used [17]. The values of color, transmittance, and opacity of the bioplastic films developed are presented in Table 6. 

**Figure 3:**
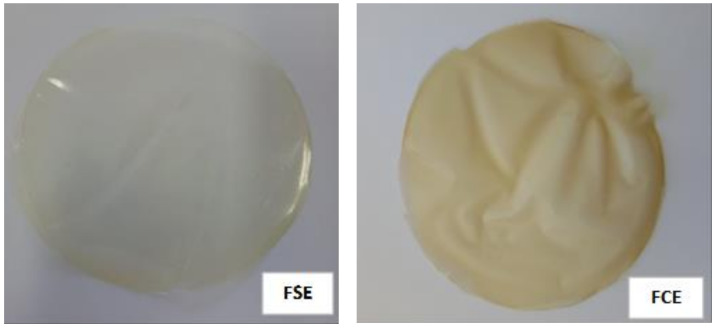
Appearance of bioplastic films, without (FSE) and with extract (FCE).

The control film was transparent and the addition of the extract decreased the transparency and consequently increased the opacity, with a slightly yellowish color (Figure 3). The total color difference indicates greater color in the film with the extract. The decrease of transmittance is a good characteristic when one intends an application as a barrier in the food area, since it protects the food from oxidation, loss of nutrients, and discoloration [48]. These observations are according to Rubilar, Candia, Cobos, Díaz, and Pedreschi [49] in films of chitosan with nanoclay and LAE (ethyl Nα-dodecanoil-l-arginate), found a decrease in transmittance in relation to the control film, and differed from Haghighi et al. [50] in films of chitosan and polyvinyl alcohol, which obtained only transparent films, regardless of the presence or not of the extract, because all showed opacity below 5. Adilah, Jamilah, Noranizan, and Hanani [51] in fish gelatin films incorporated of mango shell extract also observed an increase in the opacity of the films as the extract was added, however, they reported opacity values below those found in this study, ranging from 0.06 to 1.11 A·mm^−1^.

**Figure 4:**
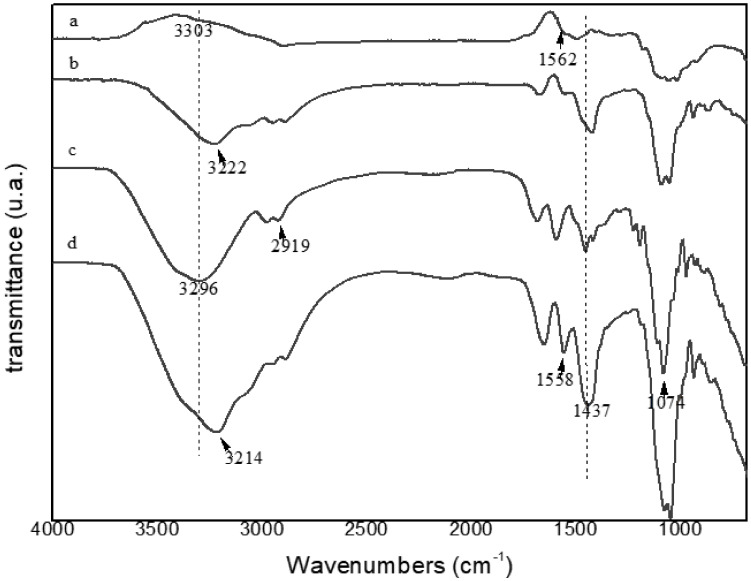
Fourier transform infrared (FTIR) spectra of (a) chitosan polymer, (b) agar-agar polymer, (c) ESF, and (d) FCE.

The FTIR spectra of the film with and without extract as well as the biopolymers (agar-agar and chitosan) were performed to determine the interactions of polymers and extract, and are shown in Figure 4.

Peaks in the range 3210–3305 cm^−1^ indicate the presence of hydroxyl (OH), however, a certain displacement between the samples is noticeable, indicating that there was an interaction of its constituents, caused by a small absorption displacement [52]. The peaks between 2900–2980 cm^−1^ were verified in all samples and refers to grouping CH [53]. The peaks around 1074 cm^−1^ present in all samples were due to the CO group [43], where again, the interaction of the compounds was noticed because the biopolymers presented lower peaks in this same wavelength, and the films without and with the extract presented more intense peaks, being more pronounced in the FCE, demonstrating the interaction of this grouping. The peak 1558 cm^−1^ expressed in FCE and chitosan corresponded to the NH connection [54]; the other samples presented a peak very close to this, probably referring to the vibration of the ester grouping (COO^−^) around the band of 1562 cm^−1^ [55]. The 1437 cm^−1^ peak present in all samples and of different intensities corresponded to the shear vibration of the CH grouping [56,57].

The surface area, volume, and pore size for the bioplastics samples with and without the extract were estimated by the BJH method (Barret, Joyner, and Halenda) and can be seen in Table 7.

The film with the extract showed a small reduction in surface area and pore size due to the interaction of biopolymers with the extract. Zhong, Zhuang, Gu, and Zhao [58] observed that when there was an increase in the molecular weight of the solution, in this case caused by the addition of the extract, the film tended to become more compact and consequently less porous, however, both samples were presented as microporous. According to Teixeira, Coutinho, and Gomes [59], sizes equal to or less than 200 Å are thus classified. Saibuatong and Phisalaphong [18] also obtained microporous films with a surface area of 15.7 m^2^g^−1^, synthesized from bacterial cellulose with the addition of 30% aloe vera. 

**Figure 5:**
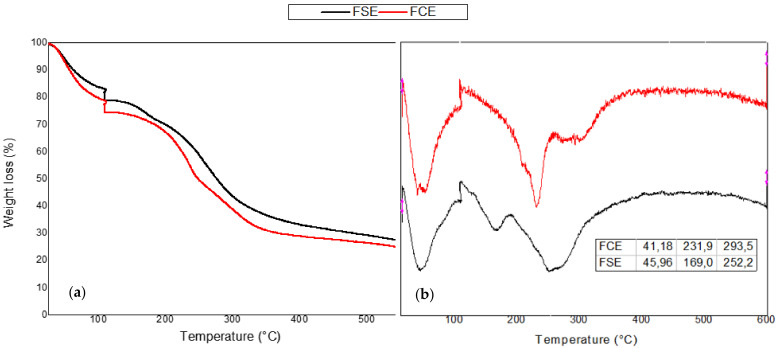
(**a**) Mass loss (%) and (**b**) thermogravimetric analysis (TGA) curve for FCE and FSE.

The results of the thermogravimetric analysis can be seen in Figure 5, where it can be seen that both films presented three main stages of degradation, with the first stage of degradation between 40 and 45 °C attributed to water evaporation, and weight loss around 10% [60]. The second stage of degradation for the FCE at a temperature of 231.9 °C corresponded to the degradation of cellulose material (agar-agar) [61]; the FSE presented a peak at 169 °C, which refers to the degradation of plasticizer (glycerol) [62], possibly the bacteriocin extract collaborated for a greater interaction of the plasticizer with the polymeric matrix, contributing to its greater thermal stability. The third stage refers to the degradation of chitosan, in a range of 252–294 °C [63]. It was also noted that the results for the control film were similar compared to the added bacteriocin extract film, corroborating the results of the mechanical properties, surface analysis, water vapor permeability, and FTIR.

When the bioplastic film has an application for a food product, it is interesting that it does not break down at the food packaging temperatures, since the degradation of the polymeric film indicates the end of the food protection, once the film is undone. In this sense, the developed film presented good thermal characteristics, being degraded at high temperatures.

Qiao, Ma, and Liu [64] reported degradation of glycerol in chitosan films at 110 to 120 °C, and in chitosan films with a fungal extract around 140 to 210°C. The same authors observed degradation of chitosan at 266 °C for the chitosan control films and 257 °C for the chitosan films plus fungal extract. 

Hankar and Rhim [42] observed a thermal degradation around 300 to 370 °C in agar composite films associated with agar degradation, since cellulosic materials have a degradation temperature in this neighborhood. Reddy and Rhim [65] reported a wider range of thermal degradation for cellulosic materials, from 200 to 370 °C, obtained on agar films and nanocellulose with papier-mâché pulp, a temperature range that was associated with agar degradation.

#### 3.2.1. In Vitro Antibacterial Analysis

The antibacterial effect of the purified bacteriocin extract and the embedded film extract can be seen in Table 8. In the film without extract, only with the presence of biopolymers (chitosan and agar-agar) and glycerol did it show no inhibition zone before the tested microbiota, which is in accordance with [66,67,68], who explained that chitosan when in film form presents negligible antimicrobial properties, because it does not diffuse in the culture medium. In this way, only the organism in direct contact with its active sites is inhibited, because it is necessary that the amino grouping present in the chitosan, which is positively charged, enters in direct contact with anionic groups present in the surface of the cell of the microorganism, so that they can react and then inhibit the synthesis of new proteins.

The absence of bacterial growth around the films or extract is characterized by a clear zone, visible to the naked eye, and called a halo of inhibition. When the microorganisms *Escherichia coli* and *Salmonella enteritidis* were analyzed, there was no significant difference (*p* < 0.05) between the bacteriocin extract and the film incorporated with it. For *Staphylococcus aureus*, the film presented a greater halo of inhibition than the extract, indicating that the extract has possibly been potentiated when entering in contact with the chitosan, showing itself more efficient in front of microorganisms with a Gram-positive membrane. The results indicate that the bacteriocin extract has antibacterial action when applied to the bioplastic film, since it maintained or increased the inhibition halos, thus confirming the efficiency of the bioplastic film developed in this study and its potential to be applied as active packaging in a food. Similar findings were reported by Sugumar, Mukherjee, and Chandrasekaran [69] found inhibition halos of 7, 11, and 15 mm for chitosan films with different concentrations of *eucalyptus* oil nano-emulsion against *Staphylococcus aureus*. Wu et al. [70] reported inhibition halos between 6 and 9 mm for *Escherichia coli* and 8 to 19 mm for *Staphylococcus aureus* in chitosan nanocomposites and Ԑ-polylisin.

**Figure 6:**
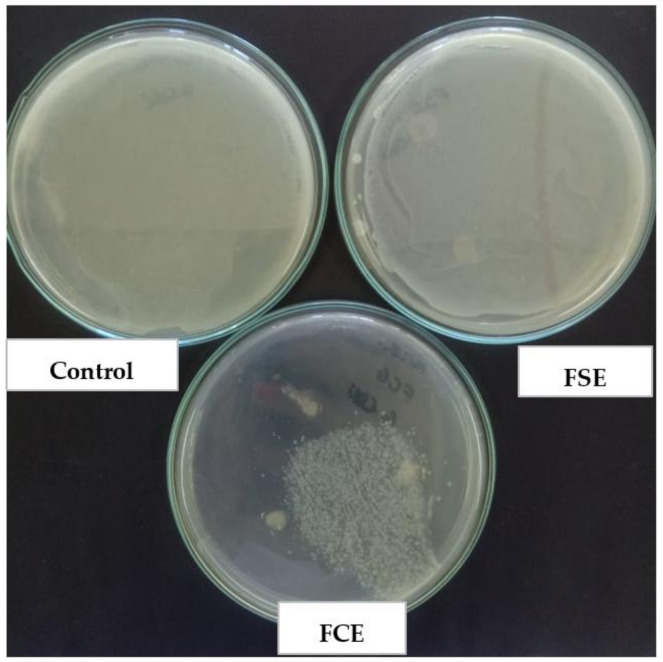
Microatmosphere test in front of *Escherichia coli.*

With the concern of real efficiency in the application as active packaging, the microatmosphere test (Figure 6) indicates that the packaging does not need to be in direct contact with the food to be effective, since the active compound has volatility and can act, even if not in direct contact. 

Figure 6 indicates the development of *E. coli,* inoculated in the Petri dish (control), and two more Petri dishes where the *E. coli* culture was sown, with the one called FSE containing the film without the incorporation of the extract and the one containing the film with the incorporation of the bacteriocin extract (FCE). It is noted that in both the control and in the FSE, there was a clouding of the culture medium, indicating the proliferation of *E. coli* after 24 h of incubation, while in the FCE, small colonies can be seen in a region of the plaque, suggesting that the volatilization of the compound occurred and was efficient in inhibiting microbial development. With this technique, the inhibitory action is due to the volatile compounds of the extract, which in the vapor phase comes into contact with microorganisms [71]. The results are in accordance with those of Kashiri et al. [72], who obtained positive effects for the microatmosphere test for *Listeria monocytogenes* and *Escherichia coli* in zeine films with the essential oil of *Zataria multiflora* Boiss; however, a greater concentration of the extracts used was necessary in comparison with disc diffusion analysis under the same conditions. Dannenberg et al. [73] obtained satisfactory results in microbial reduction of *Salmonella Typhimurium*, *Staphylococcus aureus,* and *Listeria monocytogenes* when using pink pepper essential oil as an antimicrobial in cellulose acetate film.

Thus, the developed film has potential for application in a food product as it has adequate mechanical characteristics for handling and packaging, has excellent physical and barrier properties as well as antibacterial capacity, and can act both in direct contact and by microatmosphere, thus helping in increasing the product’s lifespan.

#### 3.2.2. Release Profile

**Figure 7:**
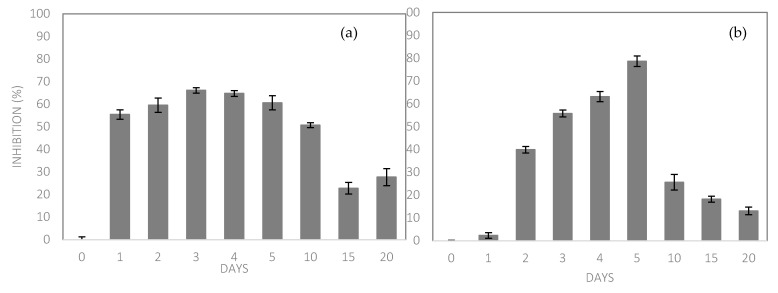
Bacteriocin release incorporated in the polymeric matrix. (**a**) Inhibition under *Escherichia coli*. (**b**) Inhibition under *Staphylococcus aureus.*

The release profile of the bacteriocin extract incorporated in the bioplastic film (aga-agar/chitosan) is illustrated in Figure 7, where it is noted that bacteriocin was gradually released, reaching the maximum release between the third and fifth day of contact with the simulant solution, where it presented 66.07 ± 1.2% of inhibition against *Escherichia coli* on the third day and 78.61 ± 2.3 under *Staphylococcus aureus* on the fifth day. The release profile was traced from the activity of the compound and not the compound itself, in this way, as the test microbiota is composed of different microorganisms, one representative of the group of Gram-positive and another of Gram-negative, the performance of the bacteriocin occurred differently due to the divergence of cell walls.

The gradual release is made possible by the characteristics of the polymeric matrix [74] mainly by solubility and porosity, which was favored in this study, since the film presented a low solubility (20.97%) and a microporous surface analysis (22.45 Å). The release of bacteriocin extract provided microbial inhibition, so the profile was elaborated in front of the inhibition that occurred in front of the microbiota test, where it was noted that during the whole time of analysis, the microbial reproduction was reduced in comparison with the control sample (time 0), which presented 100% microbial growth and in turn 0% of inhibition.

The same analysis was performed with the film without extract (FSE), and showed no signs of inhibition, indicating that the inhibition observed in the FCE came from the bacteriocin extract added to the formulation.

Roy and Rhim [52] found that in chitosan and curcumin films, curcumin is released in up to 100 min and slows down to the point of balance with the solution. Xu et al. [23], when analyzing films of gum Arabic and chitosan incorporated with essential oils of cinnamon and clove, found an initial fast release that remained constant during 600 min, in 40 min, there was a release of 58.90% of the oils and 99.42% in 300 min of contact with the same simulant solution analyzed in this study. Chen, Xiao, Cai, and Liu [22] observed a release of 60% of the tea polyphenol in 150 min of contact with the simulant solution and was kept constant for 600 min, when analyzing zein and gelatin films with polyphenol.

### 3.3. Microbiological Monitoring In Situ

The results of the microbiological follow-up of the Minas Frescal cream cheese are shown in Table 9.

It was observed that after storage, a reduction in the count was obtained including the control film, due to injuries caused by the cold, which was predicted because the cold acts in inhibiting or delaying the multiplication of microorganisms [75]. However, the film with extract (FCE) contributed to a greater reduction in the microbial load, since it showed a 53.4% reduction compared to the FSE in the same storage time. The FCE reduced 2.62 log CFU/g, while the FSE reduced 1.79 log CFU/g.

Seydim, Sarikus-Tutal, and Sogut [76], in 15 days of storage of Kasar cheeses using whey protein films with nisin as a separator, obtained a log reduction for *Escherichia coli* and *Staphylococcus aureus*. Artiga-artigas, Acevedo-Fani, and Martín-Belloso [77] also found a decrease from 6 to 4.6 log CFU/g in the count of *Staphylococcus aureus* during 15 days of storage of cheese samples coated with an edible nanoemulsion-based essential oil of oregano and tangerine fiber.

Dannenberg et al. [73], when analyzing the antibacterial action of cellulose acetate films incorporated with pink pepper essential oil in sliced mozzarella cheese, obtained a reduction from 4.30 to 2.91 log CFU/g for *Staphylococcus aureus* in 12 days of storage and found no significant action for *Escherichia coli*. Goksen, Fabra, Ekiz, and López-Rubio [78] found a reduction of 1.7 and 1.6 log CFU/g in the growth of *Staphylococcus aureus* by adding zein film with essential oil extracted from *Laurus nobilis* (bay leaf) and *Rosmarinus officinalis* (rosemary), respectively, in slices of Gouda cheese for a time of 28 days. Youssef, El-Sayed, El-Sayed, Salama, and Dufresne [79] found a reduction of 1.30 log CFU/g in the coliform group over a period of 30 days in Egyptian soft white cheese when using a bionanocomposite film of chitosan/carboxymethyl cellulose/zinc oxide.

In light of what has been reported, the importance of using this type of active packaging is noted, in order to reduce the incidence of these high contaminations. It can be seen that the bacteriocin embedded film studied in the present work has reduced from 1.1 × 10^6^ ± 1.65 × 10^5^ CFU/g for 3.7 × 10^3^ ± 4.36 × 10^2^ for *Staphylococcus aureus* and 150 ± 43.3 NPM/g for 23 ± 0.00 NPM/g for *Escherichia coli*, presenting itself as a great alternative for use in products of this origin.

## 4. Conclusions

The synthesis of the bioplastic film was successful, and the material showed characteristics required to contain, protect, and increase the shelf life of the Minas Frescal cream cheese. The film remained intact when in contact with the food, thus allowing an efficient barrier. Still, it presented a low transmittance, thus allowing a barrier to the direct incidence of light in order to maintain the sensory characteristics of the product. There was homogeneity of the matrix-forming compounds. Antibacterial responses in vitro, in situ, and microatmosphere showed the effectiveness of the active film. The bioplastic film allowed a gradual release of the bioactive compound during the contact time, allowing for an increase in the microbiological stability of the product. Thus, the bioplastic film has the potential to be used as an active form of packaging.

## Figures and Tables

**Table 1:** Real and coded values of experimental design (%).

Level	Agar-Agar (x1)	Chitosan (x2)	Glycerol (x3)
−1.68	0.0	0.0	10.0
−1	0.4	0.8	18.0
0	1.0	2.0	30.0
1	1.6	3.2	42.0
1.68	2.0	4.0	50.0

The percentage of plasticizer is expressed in relation to the total amount of polymer. The total film-forming solution was 50 mL.

**Table 2:** Matrix of the central composite rotational design (CCRD) (coded and real variables).

Treatments	Independent Variable			Dependent Variable	
	Agar-Agar (g)	Chitosan (g)	Glycerol (%m Glycerol/m Dry Polymer Mass)	TS (MPa)	EB (%)
1	−1 (0.20)	−1 (0.40)	−1 (18.09)	3.03 ± 0.74	41.10 ± 2.62
2	+1 (0.80)	−1 (0.40)	−1 (18.09)	10.36 ± 0.94	25.60 ± 2.29
3	−1 (0.20)	+1 (0.60)	−1 (18.09)	11.15 ± 2.96	30.76 ± 2.82
4	+1 (0.80)	+1 (0.60)	−1 (18.09)	6.79 ± 2.86	24.46 ± 3.48
5	−1 (0.20)	−1 (0.40)	+1 (41.09)	1.31 ± 0.19	17.99 ± 0.32
6	+1 (0.80)	−1 (0.40)	+1 (41.09)	5.63 ± 1.56	17.45 ± 2.65
7	−1 (0.20)	+1 (0.60)	+1 (41.09)	4.39 ± 1.02	34.07 ± 3.90
8	+1 (0.80)	+1 (0.60)	+1 (41.09)	7.09 ± 1.03	25.23 ± 3.20
9	−1.68 (0.00)	0 (1.00)	0 (30.00)	3.11 ± 0.18	51.04 ± 3.64
10	+1.68 (1.00)	0 (1.00)	0 (30.00)	9.47 ± 2.53	14.90 ± 2.20
11	0 (0.50)	−1.68 (0.00)	0 (30.00)	4.13 ± 0.71	26.30 ± 3.78
12	0 (0.50)	+1.68 (2.00)	0 (30.00)	4.57 ± 1.56	17.88 ± 2.77
13	0 (0.50)	0 (1.00)	−1.68 (10.00)	10.59 ± 1.84	23.37 ± 3.10
14	0 (0.50)	0 (1.00)	+1.68 (50.00)	3.54 ± 0.69	18.18 ± 3.91
15	0 (0.50)	0 (1.00)	0 (30.00)	8.88 ± 1.04	22.95 ± 1.83
16	0 (0.50)	0 (1.00)	0 (30.00)	8.08 ± 2.09	22.16 ± 3.21
17	0 (0.50)	0 (1.00)	0 (30.00)	10.467 ± 0.28	21.00 ± 1.68

TS: tensile strength at break; EB: elongation at break.

**Table 3:** Analysis of variation (ANOVA).

	SQ	GL	MQ		$F_{cal}$	$F_{tab}$	R^2^
TS							
Regression	137.87	9	15.32		4.87	2.72	0.86
Residue	22.01	7	3.14				
Total	159.88	16					
EB							
Regression	1116.53	9	124.06		3.22	2.78	0.80
Residue	269.81	7	38.544				
Total	1386.34	16					

TS: Tensile strength; EB: Elongation; SQ: Quadratic sum; GL: Degrees of freedom; MQ: Quadratic mean; and R: Determination coefficient.

**Table 4:** Tensile strength (TS) and elongation at break (EB) of films with FCE and without FSE extract.

	TS (MPa)	EB (%)
FSE	13.57 ± 2.17 ^a^	15.51 ± 2.87 ^a^
FCE	11.08 ± 2.33 ^a^	18.66 ± 2.44 ^a^

The values are presented as mean ± standard deviation. Different letters in the same column indicate significant differences (*p* < 0.05). FSE: Film without extract; FCE: Film with extract.

**Table 5:** The results of the physical, barrier, and chemical characterization for films produced with and without the extract.

	FSE	FCE
Thickness (mm)	0.048 ± 0.009 ^b^	0.099 ± 0.015 ^a^
WVP (10^−13^ kg·m^−1^·Pa^−1^·s^−1^)	1.498 ± 0.97 ^a^	2.045 ± 0.28 ^a^
Solubility (%)	10.281 ± 1.038 ^b^	20.974 ± 2.958 ^a^
Swelling property (%)	67.455 ± 0.967 ^a^	35.755 ± 3.245 ^b^
Moisture content (%)	20.936 ± 0.325 ^a^	15.852 ± 2.705 ^b^

Values are presented as mean ± standard deviation. Different letters on the same line indicate significant differences (*p* < 0.05). FSE: Film without extract; FCE: Film with extract.

**Table 6:** Measurement of color, transmittance, and opacity.

	L*	a*	b*	ΔE	Transmittance (%)	Opacity (A·mm^−1^)
**FSE**	88.49 ± 0.19 ª	0.60 ± 0.44 ^b^	−1.78 ± 1.74 ^b^	11.12 ± 0.26 ^b^	86.37 ± 1.42 ª	3.07 ± 0.59 ^b^
**FCE**	81.12 ± 1.16 ^b^	2.39 ± 0.16 ª	11.96 ± 2.25 ^a^	22.01 ± 2.21 ^a^	37.57 ± 3.81 ^b^	20.81 ± 1.27 ^a^

Values are presented as mean ± standard deviation. Different letters on the same line indicate significant differences (*p* < 0.05). ESF: Film without extract; FCE: Film with extract.

**Table 7:** Barret, Joyner, and Halenda (BJH) characterization of bioplastic films with and without the extract (FSE and FCE, respectively).

Bioplastics Films	Surface Area (m^2^g^−1^)	Pore Size (Å)
FSE	11.22	25.35
FCE	10.34	22.45

**Table 8:** Antibacterial effect (mm) of purified bacteriocin extract (EPS) and control bioplastic films (FSE) and incorporated bacteriocin (FCE) in front microbial tests.

	*Escherichia coli*	*Salmonella enteritidis*	*Staphylococcus aureus*	*Listeria monocytogenes*
EPS	13.04 ± 1.76 ^aA^	11.38 ± 0.57 ^aAB^	9.27 ± 0.015 ^bB^	-
FCE	13.57 ± 0.74 ^aA^	11.80 ± 1.39 ^aAB^	19.86 ± 0.43 ^aC^	9.91 ± 0.05 ^B^
FSE	No inhibition zone			

The values are presented as mean ± standard deviation. Different lower case letters in the same column indicate significant differences. Different upper case letters in the same line indicate significant differences (*p* < 0.05).

**Table 9:** Microbiological stability in situ.

Coagulase Positive Staphylococci (CFU/g)			
Analysis Days	FSE	FCE	
Time 0	1.1 × 10^6^ ± 1.65 × 10^5 A^		
7th Day	8.1 × 10^5^ ± 1.4 × 10^5 aB^		2.8 × 10^5^ ± 2.4 × 10^5 bB^
14th Day	4.3 × 10^4^ ± 1.5 × 10^4 aC^		1.1 × 10^4^ ± 1.0 × 10^3 bC^
21st Day	1.8 × 10^4^ ± 1.3 × 10^4 aD^		3.7 × 10^3^ ± 4.36 × 10^2 aD^
Thermotolerant coliforms (NPM/g)			
Time 0	150 ± 43.3 ^A^		
7th Day	93 ± 0.0 ^aB^		43 ± 2.89 ^bB^
14th Day	75 ± 16.26 ^aC^		43 ± 0.00 ^bB^
21st Day	43 ± 0.00 ^aD^		23 ± 0.00 ^bC^

The values are presented as mean ± standard deviation. Different lower case letters in the same column indicate significant differences, different upper case letters in the same line indicate significant differences (*p* < 0.05).
